# Recommendations to improve imaging and analysis of brain lesion load and atrophy in longitudinal studies of multiple sclerosis

**DOI:** 10.1007/s00415-012-6762-5

**Published:** 2012-12-21

**Authors:** H. Vrenken, M. Jenkinson, M. A. Horsfield, M. Battaglini, R. A. van Schijndel, E. Rostrup, J. J. G. Geurts, E. Fisher, A. Zijdenbos, J. Ashburner, D. H. Miller, M. Filippi, F. Fazekas, M. Rovaris, A. Rovira, F. Barkhof, N. de Stefano, MAGNIMS Study Group

**Affiliations:** 1 Department of Radiology, VU University Medical Center, Amsterdam, The Netherlands; 17 Department of Physics and Medical Technology, VU University Medical Center, Amsterdam, The Netherlands; 2 Oxford Centre for Functional Magnetic Resonance Imaging of the Brain, Oxford University, Oxford, UK; 3 Department of Cardiovascular Sciences, University of Leicester, Leicester Royal Infirmary, Leicester, UK; 4 Department of Neurological and Behavioral Sciences, University of Siena, Siena, Italy; 5 Image Analysis Center and Department of Radiology, VU University Medical Center, Amsterdam, The Netherlands; 6 Centre for Healthy Aging, University of Copenhagen, Glostrup, Denmark; 7 Department of Diagnostics, Copenhagen University Hospital, Glostrup, Denmark; 8 Department of Anatomy and Neuroscience, VU University Medical Center, Amsterdam, The Netherlands; 9 Department of Biomedical Engineering, Lerner Research Institute, Cleveland Clinic, Cleveland, Ohio USA; 10 Biospective, Inc., Montreal, Quebec Canada; 11 Wellcome Trust Centre for Neuroimaging, London, UK; 12 Department of Neuroinflammation, Queen Square Multiple Sclerosis Centre, UCL Institute of Neurology, London, UK; 13 Neuroimaging Research Unit, Institute of Experimental Neurology, Division of Neuroscience, San Raffaele Scientific Institute, Vita-Salute San Raffaele University, Milan, Italy; 14 Department of Neurology, Medical University Graz, Graz, Austria; 15 U.O. Riabilitazione Neuromotoria - Centro Sclerosi Multipla, IRCCS S. Maria Nascente - Fondazione Don Gnocchi, Milan, Italy; 16 MR Unit (Deparment of Radiology), Hospital Universitari Vall d’Hebron, Barcelona, Spain

**Keywords:** Magnetic resonance imaging, Multiple sclerosis, Brain atrophy, White matter lesions, Image analysis

## Abstract

Focal lesions and brain atrophy are the most extensively studied aspects of multiple sclerosis (MS), but the image acquisition and analysis techniques used can be further improved, especially those for studying within-patient changes of lesion load and atrophy longitudinally. Improved accuracy and sensitivity will reduce the numbers of patients required to detect a given treatment effect in a trial, and ultimately, will allow reliable characterization of individual patients for personalized treatment. Based on open issues in the field of MS research, and the current state of the art in magnetic resonance image analysis methods for assessing brain lesion load and atrophy, this paper makes recommendations to improve these measures for longitudinal studies of MS. Briefly, they are (1) images should be acquired using 3D pulse sequences, with near-isotropic spatial resolution and multiple image contrasts to allow more comprehensive analyses of lesion load and atrophy, across timepoints. Image artifacts need special attention given their effects on image analysis results. (2) Automated image segmentation methods integrating the assessment of lesion load and atrophy are desirable. (3) A standard dataset with benchmark results should be set up to facilitate development, calibration, and objective evaluation of image analysis methods for MS.

## Introduction

Longitudinal magnetic resonance imaging (MRI) studies of focal brain lesions and brain atrophy play an important role in the study of multiple sclerosis (MS) in that they help to improve understanding of disease pathobiology [1] and its clinical and cognitive effects [2], and to investigate the effect of therapeutic strategies [3, 4]. MRI assessment of lesion burden and of volumetric changes in the brain cover both the focal and diffuse aspects of the underlying pathological processes and can be achieved using standard structural imaging pulse sequences. There are, however, several limitations in their application to the study of MS. In the brain, while white matter (WM) lesions can easily be detected using standard proton density (PD)/T2-weighted or FLAIR imaging, the detection of focal gray matter (GM) lesions by standard sequences is much less reliable [5], due to the different tissue composition and pathological substrates [1]. Regarding brain atrophy, volumetric measures are sensitive to MS-related changes due to neuroaxonal loss, gliosis, demyelination, and possibly remyelination, but are also influenced by many other biological factors such as the degree of edema and hydration status of the tissues (e.g., [6–8]). In addition, while image acquisition techniques have already been standardized to a large degree, the image analysis methods needed to obtain reliable measures are not yet standardized and yield variable results [6, 9]. A recent review of the literature on correlative studies between MRI and histopathology in MS [10] recommended improvement of imaging specificity, high-resolution image acquisition, and use of combination of imaging methods in longitudinal studies to gain a deeper understanding of the disease processes in MS.

Against this background, this paper focuses on what we consider desirable future developments in image acquisition and analysis for the longitudinal assessment of brain lesions and brain atrophy in MS. Previous guidelines formulated in 1998 [11] regarding quantitative MR image analysis in MS served as the background against which we examined the current state of the art to derive recommendations on the development and application of image analysis methods for optimal assessment of brain lesions and atrophy in MS. Other important issues, such as the assessment of lesions and atrophy in the spinal cord and optic nerve, as well as the heterogeneity of focal lesions and the patterns of tissue changes in the normal-appearing brain, were excluded from this paper since, with the possible exception of spinal cord abnormalities, all these investigations require more advanced imaging techniques, hampering large-scale implementation. In the following sections, for each topic we will first present a brief position statement followed by the reasoning behind it.

## Image acquisition

### Position statement

Image acquisition should use isotropic 3D pulse sequences with multiple image contrasts to improve and extend analyses of lesions and atrophy, across timepoints. Image artifacts need special attention since they have significant effects on image analysis results.

### Reasoning

#### Increasing sensitivity for detecting and quantifying lesion and atrophy changes

While 2D (i.e., multislice) imaging has certainly proven its value in diagnosis and research, it is equally clear that with through-plane spatial resolutions on the order of 3 mm, the sensitivity for detecting localized subtle tissue changes over time is limited. One problem that may occur is subject motion between the acquisition of two interleaved sets of slices, which can introduce substantial errors in lesion load measurement as recently described [12]. However, even when interleaved scanning-related problems are absent or can be resolved, 2D imaging introduces severe limitations. Not only do images with anisotropic spatial resolution contain no information about the spatial distribution of signal through the slice, but any co-registration between images from different timepoints will inevitably introduce interpolation artifacts. This is not only the case if repositioning is poor, but even when previously published repositioning guidelines [11] are followed. For example, in cases where there is substantial atrophy between scans, good repositioning of the MR slices cannot circumvent the need to deform the image in the through-plane direction to match the brain, which introduces interpolation errors. Conversely, 3D acquisition schemes offer the advantage of allowing improved through-plane spatial resolution. This leads to improved image registration, and also to smaller interpolation-induced resampling errors, compared to 2D images with thick slices. It is now feasible on most scanners to acquire 3D image datasets with (near) isotropic resolution in clinically acceptable scan times [13].

It is more practical to acquire images using 3D pulse sequences with T1 weighting than with T2 weighting, and T1-weighted 3D images have become the standard imaging method for the study of brain atrophy. The application of other contrasts with 3D imaging in practical scan times is being made possible through recent efforts combining 3 Tesla scanners, with phased-array receiver coils, parallel acquisition, and a variable flip angle scheme [14]. Such developments are crucial to the study of focal WM and GM lesions with resolution comparable to that achievable in T1-weighted imaging. It is likely that the assessment of T2 lesion volume change, which is now an outcome measure in many clinical trials of new putative treatments for MS (e.g., [15, 16]), will be more accurate when this improved spatial resolution is employed. In addition, several techniques can be tailored to the imaging of GM lesions (DIR, MP-RAGE, SPGR, PSIR) and can be implemented with 3D acquisition; these are being systematically evaluated [5, 13, 17–23].

Longitudinal group studies looking for subtle, localized changes in lesions would also benefit from high-resolution 3D acquisition. One example application is the group-level lesion probability mapping (LPM) approach, which several studies have used cross-sectionally [24–29], but few so far have used to investigate longitudinal changes [30, 31]. Another example is the assessment of localized lesional change in individual patients through subtraction imaging, which has been shown to be improved when using near-isotropic spatial-resolution 3D imaging compared to 2D imaging. Using 3D images, more active lesions were detected, and inter-rater reliability was greater than for 2D images [32]. It should be expected that detecting within-patient changes and establishing relations at a group level would be more sensitive to minor differences if better spatial resolution is employed. Finally, the spatial, temporal, and possibly causal relations between brain lesions and brain atrophy [30, 33, 34], could be studied better if both the atrophy and the lesions were imaged with (near) isotropic spatial resolution of the order of 1 mm.

It would clearly be advantageous to define a set of 3D imaging techniques with multiple contrasts that capture as many of the known aspects of brain changes in MS as possible in both the GM and WM, with optimized image contrast and good spatial resolution.

#### Image artifacts

Care must be taken to minimize image artifacts, which can have a large influence on the results from image analysis. Common artifacts include radiofrequency (RF) intensity non-uniformity, phase-encode ghosting, signal wrap, and geometric distortion due to gradient non-uniformity, and B_0_ inhomogeneity. A relevant review can be found here [35].

Radiofrequency non-uniformity, which results in slow spatial variations of signal intensity known as the bias field, is usually partially corrected during image acquisition, although the increasingly common use of coil arrays and very high field scanners has led to an increase in the prevalence, severity, and variability of bias field in most images. Image analysis methods should include a bias field correction if necessary, as is commonly done in tissue-type segmentation methods [36, 37] or using stand-alone correction methods such as N3 [38].

Phase-encode ghosting artifacts are due to a mismatch between the true phase of spins and the phase corresponding to their spatial position; the signal from these spins can then erroneously appear elsewhere in the image. They arise when a collection of spins moves between phase encoding and read-out, either through motion of the entire head (motion artifacts) or flow of blood or CSF (flow artifacts). Motion of the head should obviously be restricted as much as possible. Blood flow artifacts may yield substantial distortions of the signal along the phase-encode direction(s), hampering analysis of those regions. Flow artifacts due to blood may be diminished by using a pre-saturation slab on the neck to minimize signal emanating from the blood entering the head. Flow artifacts, which increase with gadolinium injection particularly at the posterior fossa, can also be minimized by reduction of phase shifts with flow compensation or gradient moment nulling, but with the penalty of increasing the echo time.

The wrap-around artifact is most frequently observed in a sagittally or coronally oriented 3D acquisition with tight planning of the volume in the anterior–posterior direction, where the nose wraps into the occipital lobe or cerebellum. However, for multiarray coils with parallel imaging, such artifacts can also occur within the brain. To allow whole-brain analyses, wrap-around artifacts, if unavoidable, should be kept outside brain voxels. For methods requiring information outside the brain, such as SPM-VBM, it may also be necessary for the subcutaneous fat and skin to be kept clear of such artifacts. This can be achieved by proper choice of the read-out direction and field-of-view, albeit probably sometimes at the cost of increased scan time.

Non-uniformity of imaging gradients gives rise to geometric distortion, due to a violation of the assumption of a linear relation between field strength and true spatial position. When uncorrected, this has been shown to substantially affect whole-brain and local atrophy rate measurement [39, 40]. The correction to remove the geometric distortion [41] does come at the cost of an additional image interpolation step which affects all subsequent analyses (although some recent, so far unpublished work has been done to combine this with other interpolations into a single step), but even so, for some analysis software (e.g., SIENA [42]) the beneficial effects of removing the distortion are greater than the potential loss of accuracy due to this additional interpolation [40].

B_0_ inhomogeneities also give rise to geometric distortion, but additionally cause signal intensity loss due to more rapid dephasing. Signal loss can be minimized by using spin–echo pulse sequences with high receiver bandwidths (high gradient strength); by acquiring for each slice a pair of images with opposite polarity gradients; or by applying a post hoc correction based on direct measurement of a B_0_ map. However, it is worth noting that B_0_ inhomogeneities are not normally significant for the type of images under consideration here, except in very high field strength scanners or with pulse sequences with long gradient echo train lengths.

For completeness, we should also consider poor SNR and tissue contrast as obvious factors influencing poor image analysis outcomes. Optimizing SNR and tissue contrast through choice of field strength, pulse sequence design, and optimization of sequence parameters prior to initiation of a study is imperative. Table 1 lists these artifacts and possible solutions.

**Table 1:** Image artifacts and possible solutions

Image artifact	Possible solution	Limitation or negative effect of proposed solution
Spatial signal intensity variation due to RF field inhomogeneity (bias field)	Measure RF bias field at acquisition Parallel transmission becoming available on latest generation of scanners Correct using bias field correction algorithm	–
Wrap-around artifacts	Change read-out direction and field-of-view	Probable increase in acquisition duration
Ghosting artifacts (motion)	Make patient as comfortable as possible Limit duration of acquisition	Too short an acquisition will lead to unacceptably low signal-to-noise ratio
Ghosting artifacts (blood and CSF flow)	Apply pre-saturation slab on neck, or perform flow- compensated acquisition	Effects on acquisition duration and outcome measures to be evaluated
Geometric distortion due to gradient non-uniformity	Correct using existing algorithms	Additional interpolation; for SIENA analysis, any negative effects seem to be outweighed by benefits
B_0_ inhomogeneity (usually limited)	Shorten TE and use higher strength imaging gradients Apply post hoc correction	Not always possible; can compromise signal-to-noise ratio Additional measurement of B0 required
Poor SNR and/or tissue contrast	Optimize pulse sequence design and parameter values	SNR increase at unchanged resolution may lead to increased acquisition duration; trade-off to be made

## Image analysis

### Position statement

Improved automated image segmentation is needed to overcome the limitations of existing methods. They should be directed toward providing an integrated assessment of lesions and atrophy.

### Reasoning

#### Standardizing lesions and atrophy measurement

Volumetric quantification of the changes in lesion load and cerebral atrophy depends crucially on tissue-type segmentation, which is influenced by both acquisition- and disease-related factors. Focusing on the disease-related factors, several recent studies have shown that the extent of WM lesions can influence GM atrophy measurements because WM lesions have MR properties similar to those of GM [43–48]. An interesting approach that has been proposed to counter this problem is lesion inpainting, whereby signal intensities of lesion voxels are substituted with those observed in normal-appearing WM, prior to further analysis [43, 45, 48]. Although this appears to be a promising approach yielding seemingly improved atrophy measurements [44, 46], the effect of the correction may change with the lesion load and the specific algorithms used for correction and segmentation. For the FAST segmentation software from FSL [37], the choice of partial volume modeling algorithm utilized by the segmentation method was shown to exert a clear influence [43]. An obvious limitation of the lesion inpainting approach is that the lesion voxels still have to be identified and correctly segmented before new intensities can be assigned to them prior to GM segmentation.

Ideally, however, tissue segmentation methods for longitudinal studies of MS should tackle these issues automatically, and we recommend that this should be done by concurrently analyzing all tissue classes. Indeed, an attempt at integrated segmentation including both lesion and atrophy assessments for a single timepoint has already been reported [47, 49]. The inclusion of all timepoints available for a patient in a single segmentation process is another step that might improve quantification. Such concurrent analysis of multiple timepoints for one patient has been implemented in the CLADA software for longitudinal cortical atrophy measurement [50] and in the FreeSurfer software package for cortical thickness measurement and deep GM volumetry [51], while another paper demonstrated how difference images, obtained by subtracting co-registered images from two timepoints, may be used in the automated quantification of lesion volume change [52].

Development of this type of integrated analysis may take substantial amounts of time, and not all issues may be solvable. It would therefore be prudent to investigate alternative approaches; such approaches could be informed by a detailed analysis of the errors that occur when applying current methods to data already collected in longitudinal studies of MS. While the “holy grail” of a comprehensive segmentation method accessible by all researchers in the field should still be pursued, improvement of existing techniques may be a useful alternative approach.

#### Most frequent sources of errors

Errors in image analysis in MS studies can be grouped into two main categories: poor registration quality, and poor tissue segmentation. In many analyses, the final tissue segmentation is preceded by an algorithm to (approximately) find the intracranial cavity [53, 54]; in that case, a third category is the incorrect inclusion of extracranial tissue in the final segmentation. Errors in each of these categories are often the result of one of a few main causes:

- pathological changes, such as severe atrophy or large WM lesion load;
- image acquisition-related factors, such as incomplete head coverage, inadequate spatial resolution (leading to substantial partial volume effects), poor tissue contrast, limited SNR, and artifacts;
- inherent limitations of the algorithm, possibly aggravated by image acquisition-related factors.

Beyond the obvious (partial) solutions of both optimizing the image acquisition for the desired analysis (e.g., using full-head coverage whenever possible), and optimizing the analysis algorithms, there are several additional steps that allow relatively easy correction or prevention of such errors, which give substantial improvements to the quality of the analyses. For group studies, registration errors due to the presence of severe pathology may be limited by using disease group-specific templates rather than standard healthy control templates, together with appropriate regularization of the registration [55]. However, when there are large pathological changes within a single patient, adequate non-linear matching between timepoints remains challenging. Errors in segmentation may be limited by using information from more than one image type, ideally in an integrated segmentation approach as recommended above. For both these issues, challenges remain, and solving both might be facilitated by the standardized test dataset discussed under recommendation (3).

Progress has recently been made in the initial segmentation of the intracranial cavity, often referred to as “brain extraction”. Brain extraction is often imperfect, leaving tissue around the eyes and optic nerves, or removing part of the brain tissue, thus potentially introducing large errors in atrophy measurements by tools that rely on the brain extraction accuracy. A previous study showed that for 2D images, manual correction of the brain extraction used by SIENA (BET) increases sensitivity to disease effects in MS [56], but this solution is not feasible for high-resolution 3D images due to the high workload that would be generated. In this case, the brain extraction option settings should be optimized until the best compromise in brain extraction is obtained across all the images to be analyzed. However, a recent paper showed that a single combination of option settings yielded quantitatively very good results across a range of 3D T1-weighted image types in MS patients [57], obviating the need for further adjustment.

#### Lesions

Many lesion segmentation algorithms have been proposed, and a useful recent review is given in [58]. We restrict the scope here to fully automated methods and those that require minimal user intervention. The methods are based on several different principles such as intensity thresholding (e.g., [59, 60]), intensity gradient features (e.g., [61]), intensity histogram modeling of the expected tissue classes (e.g., [49, 62, 63]), identification of nearest neighbors (from a training data set) in a feature space (e.g., [64–66]), or fuzzy connectedness (e.g., [67, 68]), often using several of these in combination. In some cases spatial (anatomical) information is included in addition to intensities (e.g., [49, 67, 69]). Algorithmic approaches to segmentation optimization include methods such as Bayesian, expectation maximization, support vector machines (e.g., [70]), k-nearest neighbor majority voting (e.g., [64, 65], and artificial neural networks (e.g., [71].

Although promising results are often reported for images from a single scanner, performance on diverse datasets can be poor due to the different tissue contrasts that may be unknown to the algorithm. This can result in large fractions of both false-positives and -negatives; these misclassifications have proved to be a barrier to widespread adoption, especially in longitudinal studies if image quality varies over time and the level of these misclassifications is inconsistent. Incorporation of “domain knowledge”, i.e., prior knowledge of the distribution of MS lesions in the brain, improves the segmentation of lesions [67], but, in our experience still does not deliver segmentations that are acceptable to researchers in the field. Because of this unreliability, practical lesion segmentation methods are generally not fully automated, and operator intervention is still needed at the level of individual lesions, usually by some form feature selection based on the local maximum intensity gradient, followed by contour following, e.g., [72–75]. Intra- and inter-observer reproducibilities of contouring are better than for manual outlining [76, 77], but the method is still labor-intensive. In order to be able to handle the large volumes of imaging data emanating from large therapeutic trials, it would seem appropriate to strive for further, if not complete, automation.

Regarding automated quantification of lesion load change, a recent review by Lladó et al. [78] highlights the state of the art and remaining challenges for application in a clinical or clinical trial setting. This review includes a table that clearly shows the lack of consistency in quantitative performance metrics used in the literature, clearly illustrating the need for standardized reporting methods. Lladó et al. classify methods for change quantification as intensity-based analysis, temporal analysis, and deformation-based analysis. An intensity-based approach to the detection of change in lesions over time could exploit a combination of registration and subtraction as used by Moraal et al. [32, 79, 80]. If an expert reviewer is available, the registration–subtraction approach allows easy identification of change, provided that the changes between timepoints due to atrophy are not too large, or a registration method is used that can deal with the resulting brain shape deformations. It was shown for 2D images that the number of changing T2 lesions observed from the beginning to the end of a trial is statistically more powerful than the number of gadolinium-enhancing lesions from monthly scans [80]. Duan et al. [52] showed the feasibility of automatically quantifying these changes in lesions from the difference images.

The methods that Lladó et al. refer to as temporal methods typically handle image series with a large number of timepoints, which is an advantage over subtraction image analysis which can only handle two timepoints at once. The method proposed by Ait-Ali et al. [81] uses expectation maximization to first estimate non-lesion tissues and then adds lesions to the model. Gerig and colleagues [82] first perform segmentation of GM and WM, and then identify active lesions based on voxel mean and variance over the course of the timepoints. Although the method by Gerig et al. leaves room for improvement, most clearly regarding between-timepoint registration (assumed to be perfect) and the model for temporal signal evolution of MS lesions (assumed to be highly similar between lesions), it does present a feasible approach to the multiple-timepoint analysis of lesions.

Deformation-based methods for lesion change quantification use the local volume change as calculated through deformable registration methods to quantify the lesion volume change. Two viable methods for lesion change quantification have been presented, i.e., that by Rey et al. [83], which is based on Thirion and Calmon [84], and that by Pieperhoff et al. [85], but both require additional modeling or operator intervention to indicate which are the lesion areas whose volume change should be quantified. The lesion segmentation problem therefore still needs to be solved in these approaches.

Three-dimensional imaging with isotropic resolution and multiple image contrasts can be expected to further increase the specificity with which change in lesions can be characterized, both in terms of their spatial location and for distinguishing and interrelating changes in different lesion types. For all these methods, there are several choices to be made on issues such as the type of registration, whether and how to include prior information on expected lesion and atrophy-related change, among others; these choices should be informed in part by comparing results against expert manual analysis.

#### Atrophy

Just as analysis of MS lesions in longitudinal studies is affected by concomitant atrophy, so too does atrophy quantification deteriorate when there are large changes in the lesion load. For example, large changes in atrophy or in lesion volumes may disrupt the accuracy of registration, which is used by many atrophy measurement methods [86, 87].

In normal aging and Alzheimer’s disease (AD), Smith et al. compared two whole-brain atrophy measurement techniques, i.e., (brain) boundary shift integral (BSI [88]) and SIENA directly and showed that the methods gave very comparable results [89]. Sample size calculations in RRMS showed similar sample sizes were required for BSI and SIENA [90]. Using images with simulated atrophy in AD, Camara et al. [91] confirmed the good agreement between BSI and SIENA. More recently, Durand-Dubief et al. [6] selected seven methods for measuring whole-brain atrophy and assessed their reproducibility across different MRI platforms. This study on nine patients scanned on three occasions over 1 year, each time on two MRI scanners, showed that registration-based methods, i.e., where the registration is performed within-subject between timepoints, particularly an optimized BSI method using k-mean clustering (KNBSI) and Jacobian integration, gave the best agreement of whole-brain atrophy measures between the two different MRI scanners.

Also in MS, but focusing on local change instead, Battaglini et al. [92] performed a qualitative comparison between two different methods for measuring local changes in atrophy over time. By comparing longitudinal VBM (using FSL) and the voxelwise SIENA-R method directly, in the same longitudinal image set from MS patients who were scanned twice, with a 3-year interval between the two scans, they showed that the cortical regions in which significant atrophy was observed were roughly similar, but the extent was very different. This result was perhaps to be expected based on the different mechanisms of the two methods, with VBM quantifying local GM density and its change over time, while SIENA-R measures displacement of the local brain-non-brain boundary. Nevertheless, this study demonstrates the influence that choice of analysis method has on the results. Both this difference between SIENA-R and longitudinal VBM, and the superiority of (within-subject) registration-based techniques may be explained by the design of the methods: analysis methods that analyze within-subject change over time directly, by concurrently analyzing multiple timepoints, make use of the fact that intra-subject variability is generally smaller than inter-subject variability. These inherently longitudinal methods may therefore be better at quantifying this change than methods that treat each timepoint separately.

As indicated in the section on image acquisition, results are also influenced by the choice of imaging parameters, and so tissue contrast and spatial resolution should be optimized. Nevertheless, the CLADA method proposed by Nakamura et al. [50] did achieve both accurate measurement of cortical thickness, and reliable measurement of cortical thickness change, in low-resolution 2D images that are (still) typical for clinical trials. Accuracy may also differ between local atrophy measurement techniques, as shown quantitatively by the simulated AD atrophy study by Camara et al. [91]: deviations from ground truth atrophy differed between two Jacobian integration methods. Moreover, the mean absolute deviation was up to 93 % of the ground truth volume change for hippocampus, indicating the need for further method improvement. Partly simulated image data in which the true change is known, as used in their study, may also facilitate such developments in MS, especially when based on representative images from MS patients and made widely available as recommended below.

In healthy subjects with a mean age of 56.5 years, Takao et al. [93] investigated the effect of scanner performance on whole-brain and local volume change measurement. They showed that scanner drift and inter-scanner variability can produce large apparent volumetric changes in VBM (using SPM), including both increases and decreases. In contrast, a recent paper on MS demonstrated that, following a standardized imaging protocol and identical longitudinal VBM analysis methods, the differences between centers in the longitudinal VBM changes observed in MS patients are much smaller than the disease-related changes, indicating that pooling of data from different centers may be feasible for longitudinal VBM analysis in MS [94]. These scanner effects are important issues in most large-scale studies in MS, and this discrepancy merits further investigation.

#### Available methods and proposed direction

Table 2 lists the currently available methods for lesion load and atrophy measurement. The list is restricted to those methods that are available for installing locally on the researchers’ own systems (not necessarily without charge). The merits and limitations of each method are briefly indicated. It is clear from the discussion of published methods above that far more methods have been developed than just the selection listed in Table 2 that are available for installing locally. This suggests that further improvement of MS research may be achieved by wider distribution of some of these methods. An objective evaluation of the performance of those methods should then be a first step. In order to distinguish between disease-related effects (different disease types, patient selection, follow-up durations, etc.) and method-related effects, such comparisons between analysis methods should be performed using the same common dataset(s). A database such as that proposed under recommendation (3) would facilitate such a comparison.

**Table 2:** Overview of available methods for lesion and atrophy segmentation that may be installed locally. See text for details

Method	Degree of automation	Main features	Limitations
*Lesion segmentation*			
EMS http://mirc.uzleuven.be/MedicalImageComputing/downloads/ems.php	Fully automated	Lesion segmentation using expectation maximization; distributed as add-on to SPM software package	Analyzes single timepoint
HAMMER-WML module for 3D Slicer http://www.nitrc.org/projects/hammerwml/	Fully automated	Lesion segmentation using support vector machines based on local image features	Analyzes single timepoint
Jim http://www.xinapse.com/	Semi-automated	Lesion segmentation using fuzzy connectedness; some user input required for finding thresholds	Analyzes single timepoint
Lesion-TOADS http://www.nitrc.org/projects/toads-cruise	Fully automated	Lesion segmentation using expectation maximization; distributed as plugin to MIPAV software package	Analyzes single timepoint
Lesion segmentation tool for 3D Slicer http://www.slicer.org/slicerWiki/index.php/Documentation/4.2/Extensions/LesionSegmentation	Fully automated	Lesion segmentation based on local morphometric features from T1w, T2w and Flair images	Analyzes single timepoint
LST: lesion segmentation toolbox http://dbm.neuro.uni-jena.de/software/lst/	Fully automated	Estimates WM, GM, CSF from 3DT1, then lesions from Flair	Analyzes single timepoint
SepINRIA http://www-sop.inria.fr/asclepios/software/SepINRIA/	Semi-automated	Non-lesion tissues segmented using expectation maximization, thresholds and morphological operations to identify lesions	Analyzes single timepoint
WMLS: White Matter Lesion Segmentation https://www.rad.upenn.edu/sbia/software/index.html#wmls	Fully automated	Multi-timepoint lesion segmentation from multiple input images using support vector machines and anatomical information	
*Atrophy*			
BSI http://sourceforge.net/projects/bsintegral/	Semi- automated	Whole-brain volume change measurement from scan pair	No distinction between tissue types
FIRST http://fsl.fmrib.ox.ac.uk/fsl/fslwiki/	Fully automated	Deep gray matter shape analysis and volumetry	Analyzes single timepoint
FreeSurfer http://surfer.nmr.mgh.harvard.edu/fswiki	Can be run in fully automated mode – manual editing preferred	Deep gray matter volumetry; cortical thickness measurement; concurrent analysis of multiple timepoints	Long calculation time
NiftySeg http://cmic.cs.ucl.ac.uk/home/software/	Fully automated	Cortical thickness measurement	Analyzes single timepoint
SepINRIA http://www-sop.inria.fr/asclepios/software/SepINRIA/	Fully automated	Whole-brain parenchymal fraction evolution across multiple timepoints	
SIENA http://fsl.fmrib.ox.ac.uk/fsl/fslwiki/	Fully automated	Whole-brain volume change measurement from scan pair	No distinction between tissue types
SIENA-R http://fsl.fmrib.ox.ac.uk/fsl/fslwiki/	Fully automated	Group level analysis of local brain atrophy from scan pairs	No distinction between tissue types
SPM - Longitudinal VBM http://www.fil.ion.ucl.ac.uk/spm/ VBM8 Toolbox – Longitudinal VBM with SPM8 http://dbm.neuro.uni-jena.de/vbm/download/	Fully automated	Group-level analysis of local GM/WM loss over time	Only informative at group level (general VBM restriction)
TOADS-CRUISE http://www.nitrc.org/projects/toads-cruise/	Fully automated	Cortical thickness (change) measurement; distributed as plugin to MIPAV software package	

## Standard test dataset

### Position statement

A standard dataset with benchmark results should be set up to facilitate development, calibration, and objective evaluation of image analysis methods for MS.

### Reasoning

Comparing the performance of one method for lesion load or atrophy measurement to another is difficult due to the lack of standardized representative data. Papers describing new methods do not always compare the new method to current ones, and even if they do, the test image dataset is rarely made available to the larger research community. Finally, different papers use different metrics to report performance of their algorithms. Hence, the results cannot be reproduced in detail, nor can they realistically be compared between methods.

In order to allow investigators to select an analysis method, based on an unbiased assessment of various alternatives, one possible approach is to create annotated longitudinal MR image datasets from carefully selected and representative MS patients from multiple scanners/centers. This database would consist of different subsets of images for addressing specific questions. The database should be accompanied by the framework necessary for carrying out objective and quantitative evaluation of different methods against “gold standard” expert annotations, including standards for reporting the results of those comparisons, and thereby facilitate an unbiased and transparent assessment of image analysis methods.

Several databases are available that meet some of these requirements. First, BrainWeb (http://www.bic.mni.mcgill.ca/brainweb/) [95] offers a simulated dataset in which, for a limited number of cases with MS lesions, image characteristics such as intensity spatial inhomogeneity and noise can be varied. Such data could be expanded by including a larger range of lesion volumes and degrees of atrophy in the simulated images. Inserting artificial lesions into images obtained from healthy controls is an approach followed in several papers assessing lesion segmentation or the effect of lesions on atrophy measurement. The advantages of this approach are that the effects of lesions can be studied in isolation, and that the ground truth is known. The main disadvantage is that healthy control images may not be similar to MS patients’ images in all respects; for example, the degree of brain atrophy may differ, or “dirty” WM may be present in MS patients while it is generally absent in healthy controls. Therefore, a test dataset should not be restricted to simulated images based on healthy control data, but should also include real patient data.

The image data for the MICCAI 2008 “MS lesion segmentation challenge” do consist of real patient data, derived from a relatively large set of patients; these data are still available online (http://www.ia.unc.edu/MSseg/). The website provides a test-set of images along with expert annotations, so that results of a segmentation method can be compared to the “gold standard” segmentation. The scores obtained using the different methods that have been tested are listed on the website, and new entries are still being frequently added. This is a good example of the kind of standardized test dataset that is needed for optimizing analysis methods in MS. However, some characteristics of the imaging data, such as the spatial resolution of the images, are different from those typically used in a clinical trial setting. Furthermore, in addition to the 2D-FLAIR images that form the dataset, different pulse sequence types such as 3D-FLAIR or 2D dual-echo PD/T2 are also needed to test the robustness of lesion segmentation methods, as well as images such as DIR for GM lesion segmentation.

For developing and optimizing atrophy measurement methods, 3D T1-weighted anatomical images using a pulse sequence such as MP-RAGE are required. The ADNI database for AD, mild cognitive impairment and healthy aging may serve as a good example here (http://adni.loni.ucla.edu/) [96]; it allows researchers to download and use image data, under certain conditions. ADNI has boosted the development of brain image analysis methods [97], thereby also improving MS research. The availability of two consecutively acquired MP-RAGE scans provides an opportunity to study the reproducibility of methods [98], and including similar scan-rescan data in an MS test dataset would be highly desirable. Another example is the OASIS project, which allows researchers to freely download a dataset that contains images of adults across a large age range, including demented and non-demented elderly (http://www.oasis-brains.org/, [99]). The OASIS dataset also contains short-term rescan images for reliability analyses.

Objectively quantifying the performance of lesion segmentation techniques is particularly challenging, since experts do not generally agree completely on which voxels should be considered as part of a lesion [100, 101]. Segmentation of cortical and subcortical GM presents similar problems. Derakshan et al. [9] performed an elegant comparison between six automated methods and six expert segmentations. Their study showed not only how well the automated methods performed compared to the average expert segmentations, but importantly it also highlighted the variability between experts, which should be taken into account in setting up a database. One of the first uses of the proposed database could be to investigate inter-expert variability, and possibly standardize manual outlining methods in order to improve the validation of automated methods for quantifying lesion volume change and atrophy rates in MS.

Finally, beyond providing test datasets, the utility could serve the analysis method development community even better by providing training data sets. The MICCAI MS lesion challenge has been mentioned above, and the sustained availability of those data allows further development of MS lesion segmentation methods. However, there is a real danger that without independent training data, further apparent improvements may not generalize when the methods are applied in new image datasets with different imaging characteristics. Therefore, it seems imperative that to make real progress, training data should be made available that captures the variability that is encountered in a real clinical or trial setting, not only the variability due to inter-patient differences, but also that due to the heterogeneity of scanners and imaging protocols.

## Conclusions

Data collection and analysis methods for longitudinal MR imaging studies of brain lesion load and brain atrophy in MS have proved to be of great value, but can be improved. We propose to (1) acquire images using 3D acquisition techniques with multiple contrasts and near isotropic spatial resolution; (2) integrate the segmentation of lesions and atrophy measurement; and (3) provide a standard test dataset containing both images and expert annotations for objective testing and evaluation of analysis methods. These points should prove complementary: the standard test dataset may facilitate development and improvement of the integrated segmentation techniques, which in turn would benefit from the isotropic spatial resolution of the acquisition.
